# The Association between Pre-Treatment Serum 25-Hydroxyvitamin D and Survival in Newly Diagnosed Stage IV Prostate Cancer

**DOI:** 10.1371/journal.pone.0119690

**Published:** 2015-03-16

**Authors:** Digant Gupta, Kristen Trukova, Brenten Popiel, Carolyn Lammersfeld, Pankaj G. Vashi

**Affiliations:** Cancer Treatment Centers of America (CTCA) at Midwestern Regional Medical Center, Zion, Illinois, United States of America; School of Medicine and Health Sciences, University of North Dakota, UNITED STATES

## Abstract

**Background/Aims:**

Emerging evidence in the literature suggests a positive association between serum 25-hydroxyvitamin D [25(OH)D], a standard indicator of vitamin D status, and survival in certain types of cancer. We investigated this relationship in newly diagnosed stage IV prostate cancer patients.

**Methods:**

A consecutive cohort of 125 newly diagnosed stage IV prostate cancer patients underwent a baseline serum 25(OH)D evaluation prior to receiving any treatment at our institution between January 2008 and December 2011. We used the vitamin D categories of “deficient (<20 ng/ml)”, “insufficient (20 to 32 ng/ml)”, and “sufficient (>32 ng/ml)”. Cox regression was used to evaluate the prognostic significance of serum 25(OH)D after adjusting for relevant confounders.

**Results:**

Mean age at diagnosis was 60 years. Of the 125 patients, 32 (25.6%) were deficient, 49 (39.2%) were insufficient and 44 (35.2%) were sufficient in vitamin D at the time of diagnosis. The median survival in deficient, insufficient and sufficient cohorts was 47.8, 44.0 and 52.6 months respectively (p = 0.60). On univariate analysis, four variables demonstrated a statistically significant association with survival: nutritional status, bone metastasis, corrected serum calcium and serum albumin (p<0.05 for all). On multivariate analysis, five variables demonstrated statistically significant associations with survival: hospital location, age, bone metastasis, serum albumin and corrected serum calcium (p<0.05 for all). Serum vitamin D status was not significant on either univariate or multivariate analysis.

**Conclusion:**

Contrary to previously published research, we found no significant association between pre-treatment serum 25(OH)D and survival in newly diagnosed stage IV prostate cancer patients. The lack of a significant association between serum vitamin D and survival in our study could perhaps be due to the fact that the disease was far too advanced in our patients for vitamin D levels to have any impact on prognosis.

## Introduction

Vitamin D is either produced in the skin as 7-dehydrocholesterol through ultraviolet blue (UVB) exposure or ingested from the diet as cholecalciferol (vitamin D3) or ergocalciferol (vitamin D2). It is subsequently converted in the liver to 25-hydroxyvitamin D [25(OH)D], the major circulating and storage form of vitamin D commonly used for evaluating the vitamin D status of patients [1]. Though 25(OH)D is not the active form of vitamin D, it is known to be the best indicator of vitamin D status as it accurately reflects vitamin D intake from all sources and has a half-life of two to three weeks compared to only four hours for the active form, 1-alpha-25-dihydroxyvitamin-D3 (1,25(OH)2D3) [2].

Hypovitaminosis D has been found to be associated with a variety of cancers including prostate. Identified mechanisms of action of vitamin D on prostate cancer occurrence and progression include decreased cell proliferation, inhibition of angiogenesis and apoptosis [3]. Prostate cells exhibit vitamin D receptors (VDR) and are able to convert 25(OH)D to the active form, 1,25(OH)2D3 [4]. The epidemiological literature reports that vitamin D insufficiency is associated with prostate cancer risk based on its role due to UVB exposure, dietary factors or as an endogenous entity [5]. A thorough discussion of vitamin D and prostate cancer by Schwartz further evaluates the effects of the hormonal form of vitamin D on prostate cells and future directions for vitamin D treatments for prostate cancer [6]. Despite these findings, two recent meta-analyses on vitamin D concentrations and prostate cancer found little or no effect on the risk of prostate cancer but were limited by the length of time between serum vitamin D testing and subsequent diagnosis of cancer [7,8].

Emerging evidence in the literature suggests a positive association between serum 25(OH)D and survival in several types of cancer. An analysis of Chinese gastric cancer patients in which serum was collected post-diagnosis but prior to any treatment for cancer noted a positive association between lower vitamin D concentrations (< = 20ng/mL) and poor prognosis [9]. Similarly in breast cancer, two reports reported that lower serum 25(OH)D concentrations (less than 20ng/mL and <14ng/mL, respectively) were associated with poorer overall survival. Again, statistical significance was reached only with serum samples taken prior to initiating treatment [10,11]. In colorectal cancer, studies have been more congruent. Two reports noted higher concentrations of vitamin D pre-diagnosis associated with improved survival [12,13]. Two other reports evaluated serum vitamin D at time of diagnosis or first surgery for colorectal cancer and also described a better survival rate with higher 25(OH)D concentrations [14,15]. Finally, a further interpretation of the NHANES III data specific to the cohort of African-Americans reported that vitamin D deficiency was a contributor to increased mortality in colorectal cancer [16]. A review of non-small-cell lung cancer patients published in two reports showed no effect in advanced stage (n = 294) but noted benefit of high vitamin D intake (> = 371 intl units/d) and higher serum vitamin D levels (> = 15.7ng/mL) in early stage disease (n = 447) [17,18].

Specific to prostate cancer, however, this relationship is less clear [19]. There have been 3 published studies evaluating the relationship between serum 25(OH)D and survival in prostate cancer. Tretli et al. found medium (20–30ng/mL) or high (>32ng/mL) levels of 25(OH)D to be associated with better prognosis [20]. However, they combined 137 newly diagnosed with 31 previously treated (clinically more aggressive) prostate cancer patients of all stages who previously received hormonal therapy. Fang et al. found men with the lowest 25(OH)D quartile to more likely to die of their cancer compared to those in the highest quartile. However, this association was attenuated after adjustment for cancer stage and Gleason score, suggesting that these variables may act as potential confounders of the vitamin D and survival relationship [21]. Finally, Holt et al., in a population-based cohort study of 1476 newly diagnosed prostate cancer patients of all stages, found no relationship between serum levels of 25(OH)D and the risk of recurrence/progression or mortality [22]. Collectively, these studies indicate that there is either no relationship between serum vitamin D and survival in prostate cancer, or if such a relationship exists, it might be confounded by other clinicopathological variables. We aimed to further investigate this association in a homogenous group of newly diagnosed stage IV prostate cancer patients treated at a national network of oncology hospitals.

## Methods

### Study Population

A consecutive series of 125 newly diagnosed stage IV prostate cancer patients treated at four Cancer Treatment Centers of America^®^ (CTCA) hospitals (located in Zion, IL, Philadelphia, PA, Goodyear, AZ and Tulsa, OK) between January 2008 and December 2011 was evaluated. This study was approved by the Institutional Review Board (IRB) at Cancer Treatment Centers of America^®^. The need for informed consent was waived by the IRB because there was no direct patient contact in this study. This study involved collection of existing data from patient records in such a manner that subjects cannot be identified, directly or through identifiers linked to the subjects.

### Vitamin D Measurement

Serum samples were collected within 30 days of first visit at our hospital, and prior to initiation of anticancer therapy. Serum was collected at the laboratory, packed in coolpacks and sent to the Laboratory Corporation of America (Raleigh, NC) where a chemiluminescence immune assay (CLIA, DiaSorin Liasion assay) was used to measure 25(OH)D. Serum samples were incubated with antivitamin-D coated microparticles and isoluminol derivative-conjugated 25(OH)D before measurement of chemiluminescent signals. Analysis was completed within 48 hours of collection. The DiaSorin Liasion 25(OH)D assay has been clinically validated to be comparable in accuracy and precision to the radioimmunoassay (RIA). This method uses the same particles used in the DiaSorin RIA technique. Studies have found this to be a rapid, accurate, and precise tool for the measurement of serum 25(OH)D [23,24].

### Statistical Analysis

Serum 25(OH)D was the primary independent variable of interest. Although there is debate regarding which cut points should be used, we used the categories “deficient (<20 ng/ml)”, “insufficient (20 to 32 ng/ml)”, and “sufficient (>32 ng/ml)” in accordance with previously published research in this area [10,20]. A comparison of clinical and demographic characteristics was made between the vitamin D categories using a 2sample t-test, a Mann Whitney test, or a chi-square test depending upon the underlying distribution of the variables.

Patient survival was the primary end point and defined as the time interval between the date of first serum vitamin D assessment and the date of death from any cause or the date of last contact/last known to be alive. Patients were followed prospectively until May 2014. All patients were alive at least 60 days after the date of serum collection. The Kaplan-Meier method was used to calculate survival. The log rank test statistic was used to evaluate the equality of survival distributions across the 2 serum 25(OH)D groups. Clinical, demographic and serum 25(OH)D variables were evaluated using univariate Cox proportional hazards models to determine which parameters showed individual prognostic value for survival. Multivariate Cox proportional hazards models were then performed to evaluate the independent prognostic significance of all variables that were evaluated in univariate analysis. We adjusted for the following variables in the multivariate analysis: age, ECOG performance status, body mass index (BMI), prostate specific antigen (PSA), season of blood draw, CTCA hospital, serum albumin, corrected serum calcium, bone metastasis and nutritional status as measured using Subjective Global Assessment (SGA). Corrected serum calcium was used instead of total serum calcium to allow for the change in total calcium due to the change in albumin-bound calcium. Season of blood draw was defined as winter: December-February; spring: March-May; summer: June-August, or fall: September-November. The effect of serum 25(OH)D and other variables on patient survival was expressed as hazard ratios (HRs) with 95% confidence intervals (CIs). For serum vitamin D, the “sufficient” category was defined as the reference category.

Cox regression with time-invariant covariates assumes that the ratio of hazards for any two groups remains constant in proportion over time. We checked this proportional hazards (PH) assumption using a combination of graphical and statistical testing procedures. First, we examined log-minus-log plots for categorical predictors. As a second approach, we ran an extended Cox model with time-dependent covariates for continuous predictors. Finally, a goodness-of-fit testing approach based on Schoenfeld residuals was used to evaluate the PH assumption.

Finally, to assess the possible influence of sample bias on the results, as well as to investigate the stability of the model coefficients, we performed a bias-corrected and accelerated (BCa) bootstrap resampling procedure. We generated 1000 samples, each the same size as the original data set, by random selection with replacement. Cox regression was then run separately on these 1000 samples to obtain robust estimates of the standard errors of coefficients, and hence the p values and 95% BCa CIs of the model coefficients [25]. All data were analyzed using IBM SPSS version 20.0 (IBM, Armonk, NY, USA). All analyses were two-tailed, and a difference was considered to be statistically significant if the p value was < = 0.05.

## Results

### Patient Characteristics

**Table 1** displays the baseline characteristics of our patients. The median follow-up was 31 months. At the time of this analysis, 49 (39.2%) patients had expired while 76 (60.8%) were considered censored. Only 6 patients were taking vitamin D supplements at the time of diagnosis. Of these 6 patients, 2 each were taking 5000, 2000 and 400 international units (IU) per day. At the time of diagnosis, bone was the most common site of metastasis with 74 (59.2%) people having bone involvement. 107 (85.6%) patients received chemotherapy, 98 (78.4%) received hormonal therapy and 66 (52.8%) received radiation therapy at our institution. A total of 50 (40%) patients received all 3 treatment modalities of chemotherapy, radiation therapy and hormonal therapy.

**Table 1:** Patient characteristics for the overall population as well as stratified by 2 serum vitamin D categories.

**Categorical Variables**	**Overall Population (n = 125)**	**Deficient: <20 ng/ml (n = 32)**	**Not deficient: > = 20 ng/ml (n = 93)**	**P-value comparing deficient and non-deficient groups**
***SGA***				0.17
Well-nourished	110	26 (23.6)	84 (76.4)	
Moderately malnourished	15	6 (40.0)	9 (60.0)	
***ECOG Score***				0.88
0	76	21 (27.6)	55 (72.4)	
1	30	6 (20.0)	24 (80.0)	
2	12	3 (25.0)	9 (75.0)	
3	7	2 (28.6)	5 (71.4)	
***Season of Blood Draw***				0.02*
Summer	33	5 (15.2)	28 (84.8)	
Fall	30	9 (30.0)	21 (70.0)	
Winter	26	3 (11.5)	23 (88.5)	
Spring	36	15 (41.7)	21 (58.3)	
***CTCA Hospital***				0.48
Philadelphia, PA	20	4 (20.0)	16 (80.0)	
Zion, IL	72	22 (30.6)	50 (69.4)	
Tulsa, OK	25	4 (16.0)	21 (84.0)	
Goodyear, AZ	8	2 (25.0)	6 (75.0)	
***PSA***				0.24
< median, 18.2	62	13 (21%)	49 (79%)	
> = median, 18.2	63	19 (30.2%)	44 (69.8%)	
***Bone Metastasis***				0.20
No	51	10 (19.6%)	41 (80.4%)	
Yes	74	22 (29.7%)	52 (70.3%)	
**Continuous Variables**	**Overall Population (n = 125)**	**Deficient: <20 ng/ml (n = 32)**	**Not deficient: > = 20 ng/ml (n = 93)**	**P-value**
Mean age (years)	60	59.2	60.3	0.52
Mean BMI (kg/m2)	29.4	30.5	29.0	0.16
Mean serum corrected calcium (mg/dl)	9.2	9.2	9.2	0.91
Mean serum albumin (g/dl)	4	3.8	4.0	0.05*
Mean PSA (ng/ml)	91.4	133.5	76.9	0.32

(SGA = Subjective Global Assessment, ECOG = Eastern Cooperative Oncology Group, CTCA = Cancer Treatment Centers of America, PA = Pennsylvania, IL = Illinois, OK = Oklahoma, AZ = Arizona, PSA = Prostate Specific Antigen, BMI = Body Mass Index, g/dl = grams per deciliter, ng/ml = nanograms per milliliter, kg/m2 = kilograms per meter squared, mg/dl = milligrams per deciliter)

*P < = 0.05

### Predictors of Vitamin D Status

**Table 1** also describes the patient characteristics stratified by the 2 categories of serum vitamin D (deficient and not deficient). Season of blood draw and serum albumin were the two variables of statistical significance. Patients diagnosed in the summer months were less likely to be deficient in vitamin D compared to those diagnosed in fall and spring. Interestingly, patients diagnosed in the winter months also had lower prevalence of vitamin D deficiency compared to those diagnosed in fall and spring. Patients with vitamin D deficiency had lower serum albumin levels compared to those with non-deficient serum vitamin D levels (p = 0.05). Well-nourished patients had a lower prevalence of vitamin D deficiency compared to malnourished patients, although the finding was not statistically significant (p = 0.17). Patients with vitamin D deficiency had greater BMI compared to those with non-deficient serum vitamin D levels, however, the trend did not attain statistical significance (p = 0.16).

### Median Survival

**Fig 1:**
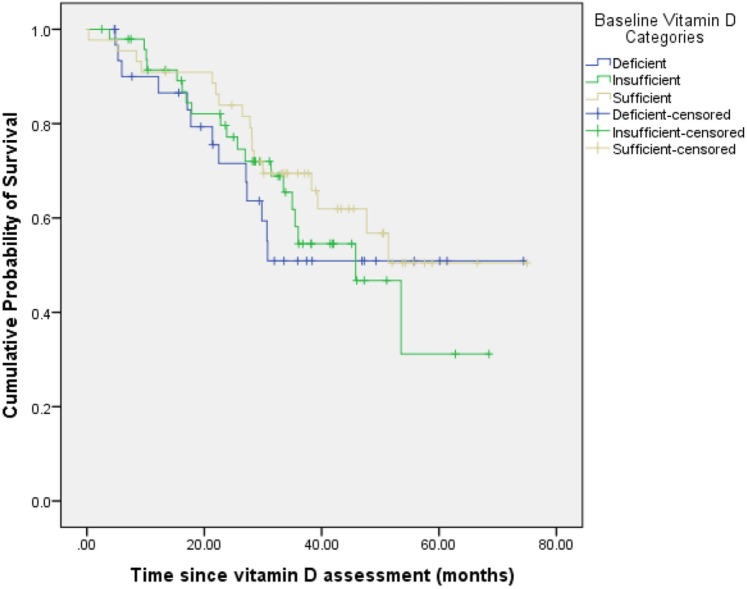
Overall survival stratified by baseline serum 25(OH)D categories. There was no statistically significant difference in the median survival times across the 3 categories of serum vitamin D.

**Table 2** shows the median survival times as a function of various clinical and demographic variables. There was no statistically significant difference in the median survival times across the 3 categories of serum vitamin D, as displayed in **Fig. 1**. Well-nourished patients had a significantly greater median survival than malnourished patients. Patients with bone metastasis had a significantly worse median survival than those without.

**Table 2:** Median survival as a function of patient characteristics.

Categorical Variables	Median Survival in Months	95% Confidence Interval	P-value
***Serum Vitamin D***			0.60
>32 ng/ml	52.6	44.4–60.8	
20–32 ng/ml	44.0	36.4–51.7	
<20 ng/ml	47.8	37.2–58.4	
***SGA***			0.04*
Well-nourished	51.2	45.5–57.0	
Moderately malnourished	31.8	22.8–40.7	
***ECOG Score***			0.13
0	52.1	45.4–58.9	
1–3	41.7	34.6–48.8	
***Season of Blood Draw***			0.85
Summer	40.8	34.2–47.4	
Fall	43.9	33.5–54.2	
Winter	53.8	42.9–64.7	
Spring	48.0	40.5–55.4	
***CTCA Hospital***			0.36
Philadelphia, PA	35.8	28.6–42.9	
Zion, IL	53.0	46.1–59.8	
Tulsa, OK	44.7	36.0–53.3	
Goodyear, AZ	23.5	16.2–30.8	
***PSA (ng/ml)***			0.19
< median, 18.2	49.2	42.8–55.7	
> = median, 18.2	45.9	38.5–53.4	
***Bone Metastasis***			<0.001*
No	65.2	58.9–71.4	
Yes	36.4	31.6–41.3	

(SGA = Subjective Global Assessment, ECOG = Eastern Cooperative Oncology Group, CTCA = Cancer Treatment Centers of America, PA = Pennsylvania, IL = Illinois, OK = Oklahoma, AZ = Arizona, PSA = Prostate Specific Antigen, ng/ml = nanograms per milliliter)

*P < = 0.05

### Univariate and Multivariate Survival Analysis

**Table 3** summarizes the results of univariate and multivariate Cox regression analyses. In the univariate analysis, each predictor was tested in isolation for its association with survival. Every 1 mg/dl increase in corrected serum calcium was associated with a 56% reduction in mortality hazard (HR = 0.44; p = 0.01) and every 1 g/dl increase in serum albumin was associated with a 42% reduction in mortality hazard (HR = 0.58; p = 0.05). Bone metastasis and nutritional status were also significantly associated with survival such that patients with bone metastasis and those who were malnourished had significantly worse survival compared to those with no bone metastasis and those who were well-nourished respectively.

**Table 3:** Univariate and multivariate Cox regression analyses of the relationship between serum vitamin D and survival.

Variables	Univariate Model HR (95% CI)	Full Model HR (95% CI)	Final Model HR (95% CI)
***Serum Vitamin D***			
>32 ng/ml (reference)			
20–32 ng/ml	1.3 (0.7–2.5)	1.1 (0.5–2.4)	
<20 ng/ml	1.4 (0.7–2.9)	1.4 (0.6–3.3)	
***SGA***			
Well-nourished (reference)			
Moderately malnourished	2.1 (1.1–4.2)*	1.4 (0.5–3.8)	
***ECOG Score***			
0 (reference)			
1–3	1.5 (0.9–2.7)	1.5 (0.7–3.2)	
***Season of Blood Draw***			
Summer (reference)			
Fall	1.1 (0.5–2.4)	1.0 (0.4–2.5)	
Winter	0.7 (0.3–1.7)	0.8 (0.3–2.1)	
Spring	0.9 (0.4–1.8)	0.8 (0.3–1.8)	
***CTCA Hospital***			
Philadelphia, PA (reference)			
Zion, IL	0.6 (0.3–1.4)	0.3 (0.1–0.9)*	0.4 (0.1–0.9)*
Tulsa, OK	0.9 (0.4–2.1)	0.3 (0.1–0.9)*	0.4 (0.2–1.1)
Goodyear, AZ	1.6 (0.4–6.0)	1.1 (0.2–4.8)	1.1 (0.3–4.1)
***PSA (ng/ml)***			
< median, 18.2 (reference)			
> = median, 18.2	1.5 (0.8–2.6)	1.2 (0.6–2.2)	
***Bone Metastasis***			
No (reference)			
Yes	4.6 (2.2–9.9)*	3.3 (1.5–7.6)*	3.7 (1.7–8.1)*
Age (continuous)	0.97 (0.94–1.01)	0.96 (0.92–0.99) *	0.96 (0.93–0.99)*
BMI (continuous)	0.95 (0.89–1.002)	0.96 (0.90–1.0)	
Serum vitamin D (continuous)	0.99 (0.98–1.01)		
Serum corrected calcium (continuous)	0.44 (0.23–0.83) *	0.43 (0.22–0.82) *	0.39 (0.22–0.71) *
Serum albumin (continuous)	0.58 (0.34–0.99) *	0.42 (0.21–0.85) *	0.37 (0.20–0.67) *

(SGA = Subjective Global Assessment, ECOG = Eastern Cooperative Oncology Group, CTCA = Cancer Treatment Centers of America, PA = Pennsylvania, IL = Illinois, OK = Oklahoma, AZ = Arizona, PSA = Prostate Specific Antigen, BMI = Body Mass Index, ng/ml = nanograms per milliliter, HR = Hazard Ratio, CI = Confidence Interval)

*P < = 0.05

In the full model, all variables tested in the univariate analysis were evaluated simultaneously in the same model. Five variables demonstrated statistically significant associations with survival: CTCA hospital, age, bone metastasis, serum albumin and corrected serum calcium. Patients with bone metastasis had a significantly worse survival than those without it. Every 1 mg/dl increase in corrected serum calcium was associated with a 57% reduction in mortality hazard (HR = 0.43; p = 0.01). Every 1 year increase in age was associated with a 4% reduction in mortality hazard (HR = 0.96; p = 0.03). Finally, every 1 g/dl increase in serum albumin was associated with a 58% reduction in mortality hazard (HR = 0.42; p = 0.02).

In the final model, only those 5 variables that were statistically significant in the full model were evaluated together. All 5 of them were found to be statistically significant.

In order to account for potential sampling bias and further investigate the stability of the classical multivariate Cox model reported in Table 3, we conducted a bootstrap resampling procedure based on 1000 samples. We found no significant differences in regression coefficients and their corresponding p values between the classical Cox regression and bootstrap Cox regression models.

## Discussion

The goal of this study was to investigate the association between serum 25(OH)D and survival in newly diagnosed prostate cancer patients. We found that vitamin D deficiency, defined as circulating levels of serum 25(OH)D <20 ng/ml, was not significantly associated with prostate cancer survival. The findings of our study are in contrast with the findings of positive association published in two earlier reports in prostate cancer investigating the same research question [20,21]. However, there are some noteworthy differences between those studies and our study that deserve careful attention.

Tretli et al. investigated 137 newly diagnosed in conjunction with 31 previously treated prostate cancer patients of all stages who previously received hormonal therapy [20]. In the previously treated subgroup, the serum 25(OH)D measurements were made on an average of 2.4 years after diagnosis and it is possible that the serum 25(OH)D levels could have been affected by the treatment received or by the disease itself. In the study by Fang et al., the serum vitamin D and survival association was rendered insignificant after adjustment for cancer stage and Gleason score [21]. Moreover, the serum vitamin D assessments were made up to several years before the diagnosis of prostate cancer. In our study, we focused on a homogeneous population of newly diagnosed stage IV prostate cancer thereby minimizing the possibility of potential confounding by disease stage and prior treatment history. Unlike the studies by Tretli et al. and Fang et al., we measured serum vitamin D within 30 days of patients’ first visit to our hospitals.

Our study findings are consistent with those recently published by Holt et al. who found no relationship between post-diagnostic serum levels of 25(OH)D and the risk of recurrence/progression or mortality in a large population-based cohort of prostate cancer patients [22]. The lack of a significant association between serum vitamin D and survival in our study could perhaps be due to the fact that the disease was far too advanced in our patients for vitamin D levels to have any impact on prognosis. However, in the studies by Holt et al. and Fang et al., no significant association was seen in early-stage disease either. Collectively, the results of our study considered against the backdrop of the existing literature in this area suggest that serum vitamin D levels measured either pre- or post-diagnosis might not be independently predictive of survival in prostate cancer after controlling for the most relevant potential confounders.

Although not statistically significant, we found lower serum levels of 25(OH)D to be associated with higher BMI, in accordance with previously published research [11,26–28]. Multiple mechanisms have been proposed to explain the association of obesity with hypovitaminosis D, including lack of sunlight exposure from physical inactivity [29] and sequestration of vitamin D in subcutaneous fat depots [30]. Another hypothesis suggests that alteration of the vitamin D endocrine system in obese subjects is characterized by secondary hyperparathyroidism which is associated with enhanced renal tubular reabsorption of calcium and increased circulating 1,25 (OH)2D which causes a feedback inhibition of 25(OH)D synthesis [31]. As a result, it has been proposed that BMI should be taken into account when assessing a patient’s vitamin D status and more aggressive vitamin D supplementation should be considered in obese cancer patients [28].

We found higher corrected serum calcium levels in our population to be significantly associated with better survival. Research has noted these variables to be inversely related, typically in the setting of hypercalcemia associated with bone metastases and more advanced disease [32,33]. Our mean corrected serum calcium was 9.2 mg/dl which is within the normal limits of 8.5 to 10.1 mg/dl. Only 6 patients had serum levels below 8.5 mg/dl and only 5 patients had serum levels above 10.1 mg/dl, with the remaining patients having serum levels within the normal range. As a result, we don’t believe this finding to have any major clinical relevance in our study.

Some limitations of our study are worth acknowledging. This is an association study which cannot prove causality. Reverse causality (the effect of cancer on serum vitamin D levels) is always a possibility in observational studies and cannot be ruled out with certainty [19]. In this study, serum 25(OH)D was measured only once at diagnosis which might not be reflective of vitamin D levels during cancer generation or progression. However, previous research has shown the reliability of a single serum vitamin D assessment over a 5-year period [26]. No formal sample size calculations were done prior to conducting this observational study. It is possible that the lack of statistical significance with regard to serum vitamin D and survival could be the result of a relatively small sample size with limited number of outcome events. In order to account for this limitation, we conducted a bootstrap resampling procedure and did not observe any significant differences between classic and bootstrap Cox regression estimates. The treatments received were not standardized as they would have been in a clinical trial setting. Finally, the available survival data could not distinguish between the death from prostate cancer and from other causes; therefore, we assessed the all-cause mortality instead of prostate cancer specific mortality.

There are some strengths of our study. We examined a homogeneous patient population of newly diagnosed stage IV prostate cancer which eliminates potential confounding by tumor stage and prior treatment history. We measured serum vitamin D at disease diagnosis prior to receiving any treatment which eliminates the possibility of treatment and lifestyle changes affecting serum vitamin D levels after diagnosis. We had clinical and demographic data available for all participants. Finally, we adjusted for a wide range of potential clinical and demographic confounders thereby minimizing the possibility of residual confounding. That being said, the possibility of residual confounding can never be completely ruled out in observational studies.

## Conclusion

In conclusion, we did not find any significant association between serum 25(OH)D and survival in newly diagnosed stage IV prostate cancer patients. Definitive evidence of vitamin D benefit on prostate cancer survival will require a randomized placebo-controlled trial.
